# The characteristics and risk factors of e-cigarette use among adolescents in Shanghai: A case-control study

**DOI:** 10.18332/tid/166131

**Published:** 2023-06-19

**Authors:** Suizi Zhao, Ziyan Li, Lulu Zhang, Zhiping Yu, Xixuan Zhao, Yan Li, Jingfen Zhu

**Affiliations:** 1 School of Public Health, Shanghai Jiao Tong University, Shanghai, China; 2 Department of Nutrition and Dietetics, University of North Florida, Florida, United States; 3 Center for Health Technology Assessment, China Hospital Development Institute, Shanghai Jiao Tong University, Shanghai, China

**Keywords:** adolescent, electronic cigarette, case-control study, influencing factor

## Abstract

**INTRODUCTION:**

This study was conducted to explore the characteristics and risk factors of e-cigarette use in adolescents, aiming for tobacco control and reducing e-cigarette use in this population.

**METHODS:**

Using 1:1 matching, 88 students from three vocational high schools in Shanghai were recruited to conduct a case (using e-cigarettes) - control (not using e-cigarettes) study. Group interviews and questionnaire surveys were used for this qualitative and quantitative mixed-methods study. The keywords were extracted from the interview data and analyzed by the Colaizzi seven-step method.

**RESULTS:**

The characteristics of adolescents’ use of e-cigarettes include young age at first use, consumption of a large amount, as well as smoking in discrete locations in order to hide from adults. The reasons for using e-cigarettes include curiosity and wanting to replace traditional cigarettes. The risk factors of using e-cigarettes include insufficient understanding of the harm of e-cigarettes at the individual level (The positive outcome expectancy points: Z= -3.746, p<0.001; The negative outcome expectancy points: Z= -3.882, p<0.001), peer influence at the interpersonal level (χ^2^=6.510, p<0.01), and the influence of social and environmental factors such as e-cigarette sales in the stores and WeChat Moments (p<0.05 for all associations).

**CONCLUSIONS:**

Having friends who use e-cigarettes, curiosity and sales exposure about e-cigarettes are important factors affecting the use of e-cigarettes by adolescents. It is necessary to strengthen the publicity of the potential hazards of e-cigarettes and to reduce overall usage by improving relevant laws and regulations.

## INTRODUCTION

Studies have found that traditional tobacco use among adolescents has declined, but the prevalence of e-cigarettes has risen rapidly^1^. Although the prevalence of e-cigarettes among adolescents in China is lower than that of countries in Europe and the United States, it is also on the rise^2^. The Chinese Center for Disease Control and Prevention (China CDC) reported that the percentages of middle school students who have heard of, used, and currently use e-cigarettes in 2021 were 86.6%, 16.1%, and 3.6%, respectively, which increased by 9.2%, 3.5%, and 0.8% compared with 2019 data^3,4^. The smoking behavior of adolescents is affected by individual, psychological, and social factors such as curiosity, the smoking behavior of friends or family members, and their social environment^5^. The Health Ecological Model (HEM) emphasizes that individuals’ health is affected not only by individual characteristics but also by interpersonal networks and social environment^6^. More specifically, at the individual level, the pleasant aromas and various flavors are significant reasons why e-cigarettes are popular among adolescents^7^. According to the tobacco survey conducted by China CDC^3^, the most commonly used flavor is fruity (63.8%) among middle school students who use e-cigarettes. Curiosity is also an important reason why adolescents use e-cigarettes. Surveys showed that 61.4% of Finnish adolescent e-cigarette users were trying something new^8^ and that e-cigarettes with flavor were more desired than those without flavor^9^.

At the interpersonal level, family and friends have a great impact on adolescents’ use rate of e-cigarettes. Among US high school students who use e-cigarettes, nearly 50% indicated that they have 3–4 friends who also use e-cigarettes^10^. Adolescents who smoked with friends were more likely to use e-cigarettes than their non-smoking friends^11^. One research study in South Korea showed that adolescents who were exposed to secondhand smoke at home were more likely to use e-cigarettes. At the social level, many factors, such as sales, education, and information, influence the usage of e-cigarettes. However, recently with the rapid spread of e-cigarette information, exposure to this information and the overall availability of e-cigarettes has caused an increased risk of e-cigarette use among adolescents^12^. Many studies have suggested that exposure to e-cigarettes is associated with an increased intention to try e-cigarettes^9,13^. Moreover, e-cigarette users may share e-cigarette-related information on the internet or social media, such as their favorite e-cigarette products, which further serve as advertisement^14^. Until now, although many studies have examined the factors associated with e-cigarette use among adolescents, only a few have explored the specific multi-dimensional characteristics and influencing factors, including friends, family and environmental factors. This study was based on the theoretical framework of the HEM and contributes to understanding the relevant factors of adolescents’ e-cigarette use. Through a case-control study, we could comprehensively explore the current problems of e-cigarette use among adolescents and provide a full-scale and targeted intervention to effectively prevent adolescent e-cigarette use.

## METHODS

### Data sources

From May to June 2021, we selected three vocational schools in three different districts (Huangpu, Putuo, Minhang) in Shanghai. Forty-four students who had used e-cigarettes were recruited as the ‘case group’, and 44 as ‘controls’ who did not use e-cigarettes, were matched (1:1) to cases of the same age and gender. In the matching process, we set the conditions for adolescents participating in this study, including class, gender, age, and e-cigarette use conditions. Then, eligible teenagers voluntarily signed up for the interview. Age and gender are among the most important factors in e-cigarette use, so we chose these factors to match after measuring the feasibility of the study. All students signed informed consent before the survey, which included the study objectives, procedures, potential risks, and benefits. The study was approved by the Ethics Committee of the School of Public Health, Shanghai Jiao Tong University.

### Measures

*Questionnaire*

A self-administered questionnaire was used to assess the general sociodemographic characteristics of participants (age, sex, grade, boarding situation and academic performance), factors associated with e-cigarette use (ever use or currently use), environmental exposure (defined as adolescents who have seen anyone using e-cigarettes over the past 30 days), perceptions (including two domains, positive and negative expectancy domains). The survey results were uniformly entered and analyzed after being reviewed and checked for completeness and errors.

Perceptions of e-cigarette use were assessed using the Adolescent E-Cigarette Use Outcome Expectancy Scale^15^, which includes eight entries (two dimensions: the positive outcome expectancy and the negative outcome expectancy) rated from ‘least likely’= 1 to ‘most likely’=10. Outcome expectancy research has been informed by the social cognitive theory, which posits that individuals who expect positive outcomes to result from a behavior are more likely to engage in that behavior. To examine the positive and negative consequences adolescents attribute to e-cigarette use, we reviewed qualitative and quantitative research on motivations and deterrents of e-cigarette use. Eight items were developed or adapted from earlier work to represent four positive and four negative outcome expectancy domains. The positive outcome expectancy factors included social enhancement, affect regulation, positive ‘smoking’ experience and positive sensory experience. The negative outcome expectancy factors were negative health consequences, addiction concerns, negative social consequences, and negative sensory experiences. Scores for each entry and the total points for the two dimensions were calculated.

*Semi-structured interview*

The outline of the interview was determined by Expert Investigation Method. Questions included the current situations of e-cigarette use and reasons for using (case group only), perceptions of e-cigarettes, attitudes and influence of family members and friends on e-cigarette use and information on the sale of e-cigarettes. The sale exposure was defined as having seen e-cigarettes sold in retail stores around schools or malls. Two trained investigators (one interviewer and one recorder) conducted and recorded interviews for eight participants (four participants from each group).

### Data analysis

*Statistical analysis*

The continuous data that did not conform to normal distribution were presented as median and interquartile range (IQR), and the categorical data were presented as frequency and percentage. To compare differences between the case and control groups, we used the non-parametric test for data that do not conform to the normal distribution and the χ^2^-test for categorical data. All analyses were conducted at an α level of 0.05 (two-sided). A p<0.05 was considered statistically significant. All data were analyzed using SPSS 26.0 software.

*Qualitative research*

Within 24 hours after the interviews, the audio recordings were transcribed into textual material by trained and dedicated personnel. The interview data were analyzed using the Colaizzi seven-step method: 1) Familiarization: transcribe the audio data and read the interview transcripts carefully; 2) Identifying significant statements; 3) Formulating meanings: code recurring and meaningful ideas; 4) Clustering themes: assemble the coded ideas; 5) Developing an exhaustive description: write a detailed, non-missing description; 6) Producing the fundamental structure: identify similar ideas and sublimate thematic concepts; and 7) Seeking verification of the fundamental structure: return to the study participants to verify for evidence^16^.

For this study, the themes were analyzed around the current situations of e-cigarette use, perceptions and opinions of e-cigarette use, peer influence, family use, and social media influence. The influencing factors of adolescents’ e-cigarette use from the individual, interpersonal and social levels were discussed.

## RESULTS

### Sociodemographic characteristics

There were 44 pairs (88 people in total) in the case and control groups, 35 (79.5%) boys and 9 (20.5%) girls in each group. There were 3 (6.3%) aged 19 years, 11 (22.9%) aged 18 years, 24 (50.0%) aged 17 years, and 10 (20.8%) aged 16 years. The number of resident students was 18 (40.9%) in the case group and 20 (45.4%) in the control group (χ^2^=0.185, p=0.667). The number of adolescents in the academic top 25%, average, and bottom 25% of the case group was 8 (18.2%), 22 (50.0%) and 14 (31.8%), respectively, compared to 18 (40.9%), 20 (45.5%) and 6 (13.6%) in the control group, which was statistically different (χ^2^=7.141, p=0.028).

### Characteristics of adolescents’ e-cigarette use

*Basic information on e-cigarette use*

Thirty-five (79.5%) adolescents in the case group started using e-cigarettes in their senior year of high school, and 9 (20.5%) started in junior high school and preparatory classes. Their first exposures were predominately receiving e-cigarettes from friends. Twenty-one (47.7%) adolescents kept the consumption to one cartridge every 2–3 days. The adolescents preferred using e-cigarettes in more hidden places (out of school, at home and in the bathroom), all outside the school’s supervision. They used e-cigarettes mainly at night. Thirty-six (81.8%) adolescents had used the same brand of e-cigarettes. The main factors adolescents considered when choosing the brand were a variety of pleasant flavors, high popularity and friends’ use.

*Reasons and feelings for e-cigarette use*

The main reasons why adolescents use e-cigarettes include curiosity and convenience. E-cigarettes are more portable and concealed, which can compensate for traditional tobacco’s disadvantages. In addition, most adolescents (80%) began to use e-cigarettes after accepting friends’ invitations to use e-cigarettes, while a few teenagers took the initiative to imitate their friends. About half of the adolescents would use e-cigarettes with their friends daily. Regarding the feelings about using e-cigarettes, 80% of the adolescents had positive feelings towards e-cigarettes; comfortable, fun, and good taste, were the descriptions frequently.

### Analysis of risk factors for e-cigarette use

*Individual level*

It was found that the total points of positive outcome expectancy factors were higher in the case group (16.5) than in the control group (6), and the total points of negative outcome expectancy factors were lower in the case group (15.5) than in the control group (26), which was statistically different (p<0.001). The four positive outcome expectancy factors (social enhancement, affect regulation, positive ‘smoking’ experience and positive sensory experience) and four negative outcome expectancy factors (negative health consequences, addiction concerns, negative social consequences, and negative sensory experiences) were statistically different between case and control group (p<0.05) (Table 1).

**Table 1:** Analysis of cognitive situations at the individual level among adolescents in Shanghai, China, 2021 (N=88)

*Factors*	*E-cigarette use (N=44) median (IQR)*	*Non-use (N=44) median (IQR)*	*Z*	*p*
**The positive outcome expectancy factors**				
Total points	16.5 (8.0–24.25)	6.0 (4.0–13.0)	-3.746	<0.001**
Social enhancement	2.0 (1.0–4.5)	1.0 (1.0–1.0)	-2.548	0.011*
Affect regulation	5.0 (1.0–7.0)	1.0 (1.0–4.0)	-3.453	0.001**
Positive ‘smoking’ experience	4.0 (1.0–6.5)	1.0 (1.0–1.0)	-3.584	<0.001**
Positive sensory experience	5.0 (2.0–8.0)	1.0 (1.0–5.0)	-3.058	0.002**
**The negative outcome expectancy factors**				
Total points	15.5 (8.0–22.0)	26.0 (20.0–40.0)	-3.882	<0.001**
Negative health consequences	7.5 (2.0–10.0)	10.0 (5.0–10.0)	-2.069	0.039*
Addiction concern	4.0 (1.0–8.75)	9.0 (4.0–10.0)	-3.014	0.003**
Negative sensory experience	1.0 (1.0–5.0)	5.0 (1.0–10.0)	-3.287	0.001**
Negative social consequences	2.0 (1.0–4.0)	7.0 (1.0–10.0)	-3.610	<0.001**

Perceptions of e-cigarette use were assessed using the Adolescent E-Cigarette Use Outcome Expectancy Scale^15^, which includes 8 entries (two dimensions: the positive outcome expectancy and the negative outcome expectancy) rated from ‘least likely’=1 to ‘most likely’=10. IQR: interquartile range. Significantly different at p<0.01*, and at p<0.001**.

The interview results showed that although adolescents had common characteristics of e-cigarette awareness, the descriptions of the two groups were different, with adolescents in the case group referring more to the flavor and concealability of e-cigarettes, while the control group referred to the convenience of e-cigarettes. Regarding adverse health outcomes, 87 adolescents (98.9%) believed e-cigarettes were harmful to health. However, the descriptions of the control group were more specific and detailed than the case group. As for physical illness, 26 (59.1%) in the case group and 30 (68.2%) in the control group mentioned lung damage such as lung cancer, pulmonary effusion, and pulmonary edema. Although most adolescents believed that e-cigarettes are addictive, they also agreed that e-cigarettes are more attractive than traditional tobacco because of their diverse flavors and concealability.

*Interpersonal level*

**Figure 1:**
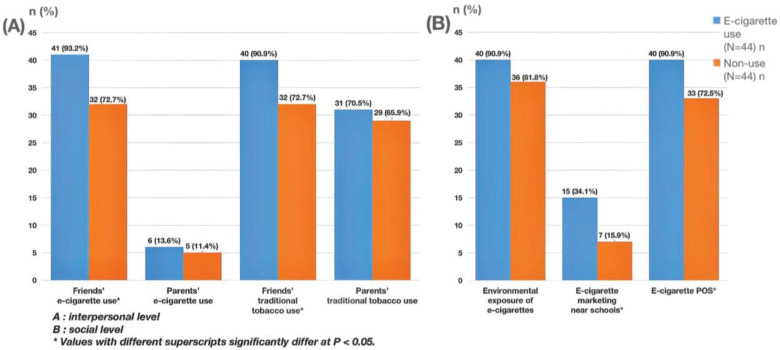
Analysis of risk factors at the interpersonal and social level among adolescents in Shanghai, China, 2021 (N=88)

The results found that the percentages of friends using e-cigarettes (93.2%) and traditional tobacco (90.9%) were greater in the case group than in the control group (72.2% and 72.7%, respectively), which were statistically different (p=0.011, p=0.027). There was no statistically significant difference between the two groups in the proportion of parents using e-cigarettes and traditional tobacco (p>0.05) (Figure 1A).

The results of the interviews showed that the majority of adolescents in the case group chose to accept cigarettes from their friends, while the attitudes of adolescents in the control group differed. Thirty-three (75.0%) in the case group chose to accept, 7 (15.9%) refused, and 4 (9.09%) said it depended on the occasion. In contrast, 2 (0.05%) adolescents in the control group chose to accept, 36 (81.8%) refused, and 6 (13.6%) said it depended on the occasion. The control group had various reasons for refusing, such as the belief that e-cigarettes were harmful to health. In terms of the attitudes toward friends’ e-cigarette use, more than 90% of adolescents said they would not consider e-cigarette use as a criterion for choosing friends.

The interviews also found that adolescents’ use of e-cigarettes influenced their attitudes toward their parents’ use of e-cigarettes. They found that parents used e-cigarettes (12.5%) less than traditional tobacco (68.2%). Twenty-three adolescents in the control group were strongly opposed to their parents’ use of traditional tobacco and e-cigarettes, but only four in the case group opposed it.

*Social level*

It was found that a higher percentage of adolescents in the case group (90.9%) had seen e-cigarettes at the point-of-sale (POS) when compared to the control group (75%) (χ^2^=3.938, p=0.047). A higher percentage of the case group (34.1%) had also seen e-cigarette marketing near the school when compared to the control group (15.9%) (χ^2^=3.879, p=0.049). There was no difference in environmental exposure to e-cigarettes between the two groups (χ^2^=1.544, p>0.05) (Figure 1B).

The interviews found that online exposure to e-cigarettes was mainly reflected on social media, especially WeChat (the most widely used social media platform in China). There were 22 (50%) adolescents in the case group who indicated that there were WeChat businesses selling e-cigarettes in their WeChat Moments (a social feature in WeChat that allows users to share texts and photos with their friends), and 6 of them reported that they had purchased e-cigarettes from them. Thirteen (29.5%) adolescents in the control group indicated that WeChat businesses were selling e-cigarettes in their WeChat Moments. A total of 40 (45.5%) adolescents reported that their friends had shared pictures or videos about e-cigarettes in their WeChat Moments. In addition, more than 80% of adolescents said that school education on e-cigarettes was limited and superficial, and the current form of education on e-cigarettes mainly was based on lectures and videos.

## DISCUSSION

This study explored the characteristics and risk factors of e-cigarette use in adolescents. The results revealed that: 1) on the individual level, the pleasant aromas and multiple flavors attracted adolescents to use e-cigarettes; 2) on the interpersonal level, friends’ e-cigarette use was a significant risk factor for e-cigarette use among adolescents; 3) on the social level, exposure of e-cigarette information online and exposure to e-cigarette sales increased the risk of e-cigarette use among adolescents. A key finding of this study was that online exposure to e-cigarettes was mainly found on social media, especially WeChat, and an increasing number of adolescents purchased e-cigarettes on WeChat businesses.

We found that 20.5% of adolescents first used e-cigarettes in junior high school and preparatory classes, and 79.5% started in high school. Previous studies have found that the average age of first use of e-cigarettes among those aged 16–24 years was 16.1 ± 2.1^17^ and 14.84 ± 1.70 years, for high school students in Canada and the US, respectively^18^. The use rate of e-cigarettes now among Chinese adolescents is much lower than that in Europe and the US, while the age of first exposure to e-cigarettes among adolescents in the case group in this study has been close to that of some European and North American countries. The reason may be that the distribution density of e-cigarette POS in developed cities such as Shanghai is much higher than in other cities and rural areas, which increases the environmental exposure to e-cigarettes and promotes the purchase and use of e-cigarettes^19^. The ability to conceal e-cigarettes compared to traditional tobacco was also one of the reasons why adolescents chose them. With the increasing regulation of the purchase and use of e-cigarettes by adolescents in recent years, there was a need to hide at schools and from parents in order to smoke. E-cigarettes have no residual tobacco smell and are easy to hide, which makes them favored by adolescents^20^.

We reviewed both qualitative and quantitative research on the negative and positive consequences that adolescents attribute to e-cigarette use. The results showed that although most adolescents were aware of the dangers of e-cigarettes, the perceptions of e-cigarettes between adolescents who used and did not use e-cigarettes were different, and the descriptions from the control group were more specific and detailed than the case group. As for specific hazards, more adolescents in the control group mentioned lung damage than in the case group, and only adolescents in the control group thought that e-cigarettes might also cause other systemic diseases. Consistently, recent studies also suggest that ENDS has adverse acute effects on cardiovascular health and respiratory health^21^ and that daily ENDS use has been shown to be associated with an increased risk of myocardial infarction^22^. There were commonalities in the positive perceptions of e-cigarettes between the two groups, such as multiple flavors, convenience, and fun. The variety of flavors available in e-cigarettes is very attractive to adolescents^23^. Noteworthy, the difference in descriptions of the two groups was that adolescents in the case group referred more to the flavor of e-cigarettes and ability to conceal them, while the control group referred to the convenience of e-cigarettes. The WHO reported^24^ that there were about 16000 flavors of e-cigarettes on the market, and most adolescents who used e-cigarettes indicated that they enjoyed a certain flavor^25^. E-cigarettes, which contained nicotine, are also addictive, but in recent years businesses have marketed them as a smoking cessation aid. However, many e-cigarette users progressed to using traditional tobacco or became dual users^26^.

Also, friends’ e-cigarette use had a significant effect on adolescents. One study found that 31.6% of adolescents reported peer influence as one of the main reasons for trying e-cigarettes^27^, and there was a higher risk for high school students to use e-cigarettes when they have friends who use them (OR=3.26)^28^. Adolescents who lacked correct perceptions of e-cigarettes were more susceptible to influences from e-cigarette users^29^. When friends used e-cigarettes or responded positively to e-cigarettes, adolescents were more likely to try e-cigarettes^30^. In addition, adolescents may be motivated to try e-cigarettes in using something new to make themselves look attractive^31^ or when e-cigarettes were seen as a social style to increase one’s attractiveness^32^.

This study also found that nearly 40% of adolescents had seen information about the sale of e-cigarettes in their WeChat Moments. WeChat businesses have gradually become a more common online sales channel for e-cigarettes. Many WeChat businesses and surrogates posted advertisements in their WeChat Moments to attract others to buy and promote them. WeChat businesses had great superiority due to the convenient transactions and fastest spread. If not regulated, WeChat businesses are likely to become the primary source of e-cigarettes for adolescents, and the availability of e-cigarettes will greatly increase the risk of using them^27^. In 2019, China issued the Notice on Further Protection of Minors from E-Cigarettes, urging e-commerce platforms, in particular, to remove e-cigarette products and withdraw related advertisements^33^, but businesses still used various methods such as renaming e-cigarettes to ‘electronic atomizers’ to avoid regulation. In addition, the price of e-cigarettes needs to be further regulated. Studies have found that^33^ increasing the price of tobacco by half would result in more health and economic benefits. In November 2021, China clarified that ‘new tobacco products such as e-cigarettes’ were regulated according to ‘the relevant regulations for cigarettes’^34^, which provided a clear legal basis for regulating e-cigarettes. But further specific implementation was needed for marketing and regulation, such as control of sales online, sales in the WeChat Moments, and sales near schools.

Family, schools and society need to make joint efforts in controlling the growing trend of adolescents’ e-cigarette use. Parents are advised not to smoke in front of their children, to quit smoking early, and to teach their children not to smoke. Results showed that schools were currently carrying out limited forms of e-cigarette control education, and the educational impact of e-cigarettes remained insufficient compared to cigarettes. Hence, targeted and diversified tobacco control education work needs to be explored. Furthermore, the country and society need to further improve the corresponding laws and regulations to fundamentally reduce the use of e-cigarettes among adolescents and create a healthy social environment.

### Limitations

This study is subject to some limitations that are worth noting. First, we used the interview research approach that allowed for deeper exploration, but the current low rate of e-cigarette use among adolescents in China and our selection of students of the same age and same gender for a case-control study resulted in a limited sample size. Second, we investigated adolescents’ memories when conducting the interviews, so they were subject to recall bias. Third, the analysis may have yet to fully account for potential confounders. Finally, this was a survey of vocational high schools in Shanghai, so the findings may not be generalizable to other regions and to adolescents not enrolled in school or public high schools.

## CONCLUSIONS

This study examined the risk factors of e-cigarette use among adolescents at three levels: individual, interpersonal, and social. Data pertinent to the sample size were extracted and analyzed using qualitative and quantitative analytic techniques. The results demonstrated that curiosity about e-cigarettes, friends’ e-cigarette use, and exposure to e-cigarette sales were significant risk factors for adolescents’ e-cigarette use, while the multiple flavors and limited e-cigarette control and inadequate education also increased the risk of e-cigarette use among adolescents. Of note, we found that WeChat businesses had gradually become a more common online sales channel for e-cigarettes, and if not regulated, they may be more likely to become the main source of e-cigarettes for adolescents. Strengthening the publicity on the harms of e-cigarettes and improving the related laws and regulations may be feasible suggestions for reducing the risk of adolescents’ e-cigarette use.

## Data Availability

The data supporting this research are available from the authors on reasonable request.
